# Erythrosin B as a New Photoswitchable Spin Label for Light-Induced Pulsed EPR Dipolar Spectroscopy

**DOI:** 10.3390/molecules27217526

**Published:** 2022-11-03

**Authors:** Arnau Bertran, Laura Morbiato, Sara Aquilia, Laura Gabbatore, Marta De Zotti, Christiane R. Timmel, Marilena Di Valentin, Alice M. Bowen

**Affiliations:** 1 Centre for Advanced Electron Spin Resonance and Inorganic Chemistry Laboratory, Department of Chemistry, University of Oxford, Oxford OX1 3QR, UK; 2 Department of Chemical Sciences, University of Padova, 35131 Padova, Italy; 3 Centro Interdipartimentale di Ricerca “Centro Studi di Economia e Tecnica dell’Energia Giorgio Levi Cases”, University of Padova, 35131 Padova, Italy; 4 The National Research Facility for Electron Paramagnetic Resonance, Department of Chemistry and Photon Science Institute, The University of Manchester, Manchester M13 9PL, UK

**Keywords:** electron paramagnetic resonance (EPR), electron spin resonance (ESR), pulsed dipolar spectroscopy (PDS), laser-induced magnetic dipole spectroscopy (LaserIMD), distance measurements, orientational effects, spin labels, photogenerated triplet state, erythrosin B, high temperature

## Abstract

We present a new photoswitchable spin label for light-induced pulsed electron paramagnetic resonance dipolar spectroscopy (LiPDS), the photoexcited triplet state of erythrosin B (EB), which is ideal for biological applications. With this label, we perform an in-depth study of the orientational effects in dipolar traces acquired using the refocused laser-induced magnetic dipole technique to obtain information on the distance and relative orientation between the EB and nitroxide labels in a rigid model peptide, in good agreement with density functional theory predictions. Additionally, we show that these orientational effects can be averaged to enable an orientation-independent analysis to determine the distance distribution. Furthermore, we demonstrate the feasibility of these experiments above liquid nitrogen temperatures, removing the need for expensive liquid helium or cryogen-free cryostats. The variety of choices in photoswitchable spin labels and the affordability of the experiments are critical for LiPDS to become a widespread methodology in structural biology.

## 1. Introduction

Electron paramagnetic resonance (EPR) pulsed dipolar spectroscopy (PDS) is a crucial tool for the study of the structure and dynamics of biomacromolecules [1,2,3,4,5]. Using microwave pulses to measure the electron–electron dipolar interaction between paramagnetic moieties, PDS techniques can be used to determine the relative distance and, for rigid systems, orientation distributions of the paramagnetic moieties, yielding information about the conformation of the biomacromolecule to which the moieties are attached [6,7,8,9,10]. Typical PDS methods, such as double electron–electron resonance (DEER) using nitroxide spin labels introduced by site-directed mutagenesis, can access the distance range of 1.5 to ca. 8 nm [11], with extension up to 16 nm possible if the biomacromolecule and solvent are fully deuterated [12]. Other stable organic radicals [13,14] and metal centers [15,16,17] are emerging as alternatives to nitroxide spin labels, offering different spectroscopic properties and improved stability for biological studies.

The photogenerated triplet state of organic chromophores has recently been introduced as a photoswitchable spin label, sparking a paradigm shift in PDS and starting the field of light-induced PDS (LiPDS) [18,19,20,21,22,23]. The formation of the EPR-active triplet states of these chromophores following photoexcitation by pulsed laser irradiation allows the study of biomacromolecules without relying exclusively on permanent paramagnetic moieties. In addition to being photoswitchable, these labels frequently lead to stronger EPR signals compared to stable spin centers, owing to the strong spin polarization arising from the initial non-Boltzmann population of the triplet state sublevels after intersystem crossing [24,25].

Several light-induced versions of PDS experiments have recently been developed based on the photogenerated triplet state of a 5(4′-carboxyphenyl)-10,15,20-triphenylporphyrin (TPP) moiety incorporated into model peptides, showing an accessible distance range similar to that of conventional PDS [18,19,26,27,28,29,30]. Light-induced DEER (LiDEER) [18] uses the photogenerated triplet formed by an initial laser flash as the detection spin while the dipolar modulation arises from the flip of a stable radical spin induced by a time-dependent microwave pulse at a second frequency. The single-frequency laser-induced magnetic dipole spectroscopy (LaserIMD) [27], on the other hand, optically switches on the dipolar interaction by forming the triplet state using a time-dependent laser pulse while detecting on a permanent radical spin. The refocused version of this technique (ReLaserIMD) offers a more accurate determination of the zero time in the dipolar time traces and is preferable to study short spin–spin distances [19]. The performances of these techniques at different microwave frequencies, X-band and Q-band, have been compared [28,31], and they have been successfully used to study the structure of different chromophore-containing proteins. A mutant of the heme protein human neuroglobin, containing a single cysteine labeled with nitroxide and reconstituted with Zn(II) proto-porphyrin IX, was studied by ReLaserIMD and a light-induced version of the relaxation-induced dipolar modulation enhancement (LiRIDME) technique, providing distance distributions in perfect agreement with high-resolution X-ray structural data [19]. The light-harvesting peridinin chlorophyll protein, containing both porphyrin and carotenoid chromophores, was bis-labeled with nitroxides and studied by LiDEER [32]. In this case, the carotenoid triplet state populated by triplet–triplet energy transfer from a nearby chlorophyl was used for detection, and triangulation with the two nitroxide radicals allowed identification of the carotene pigment involved in photoprotection. LiDEER was also employed to identify the preferential binding sites of different functionalized porphyrins to the human serum albumin protein singly labeled with nitroxide [21].

The best choice of technique to use for a particular system depends on the relative relaxation times of the different spin centers and the length of time trace that is required to measure the inter-spin interaction. The principles of LiDEER and LaserIMD are combined in light-induced triplet–triplet electron resonance spectroscopy (LITTER) [29], where photogenerated triplets are used as both detection and pump spin centers, and no permanent radical is required.

The anisotropy of EPR spectra can be exploited to obtain information on the relative orientation between the spin centers used for detection and the dipolar vector connecting the two spin centers [8]. Using TPP triplet states, the orientational effects in the dipolar traces from LiDEER, ReLaserIMD, and LITTER have been used to obtain additional information on the conformation of the molecules of study in frozen solutions [29,30]. This can be simulated and analyzed in a similar way to conventional orientationally selective DEER results [8], as all experiments involve a change in the spin state of the pumped spin of Δm_s_ = ±1.

The search for new photoswitchable spin labels for LiPDS, which could be attached to proteins that lack intrinsic photoexcitable groups, is an active area of research and a priority for the development of the field. In addition to having the right spectroscopic properties, good candidate labels for structural biology studies must be small, biocompatible, and commercially available in functionalized forms suitable for biolabeling [33]. Halogenated derivatives of fluorescein, such as eosin Y (EY) and rose Bengal (RB), and thioxanthene-based chromophores, such as ATTO Thio12 (AT), have been proposed as photoswitchable triplet labels based on spectroscopic characterization and DFT calculations [34] and were subsequently used in LaserIMD studies of the protein oxidoreductase thioredoxin in conjunction with nitroxide labels [22]. Orthogonal labeling strategies were exploited, combining the conventional maleimide-cysteine conjugation chemistry with the copper-catalyzed azide-alkyne cycloaddition between azide-functionalized labels and alkyne-bearing non-canonical amino acids. Promising results were obtained at Q-band and liquid helium temperatures. However, these studies did not take into consideration the orientational effects arising from the anisotropy of the nitroxide EPR spectrum at Q-band.

Here, we report the first utilization of erythrosin B (EB) as a photoswitchable triplet spin label for LiPDS, as previously proposed [34]. By performing multiple ReLaserIMD experiments resonant with different parts of the nitroxide spectrum, we exploit the orientational effects arising from the anisotropy of the nitroxide spectrum at Q-band to extract information both on the inter-spin distance and on the conformation of a model peptide in frozen solution. In addition, we show the feasibility of these experiments above liquid nitrogen temperatures, removing the need for expensive liquid helium or cryogen-free cryostats in LiPDS.

## 2. Results and Discussion

**Figure 1:**
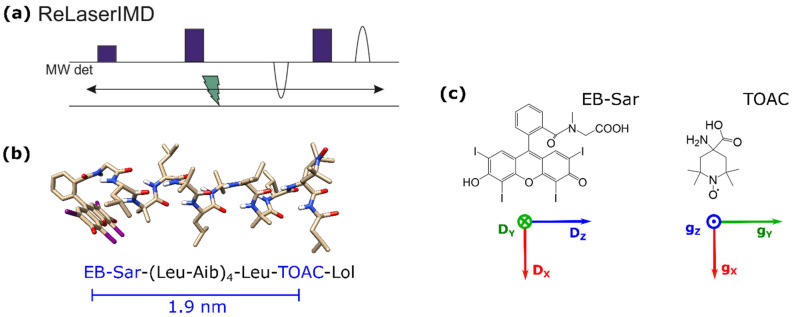
(**a**) ReLaserIMD pulse sequence. (**b**) Ground-state DFT-optimized geometry of **1** and corresponding amino acid sequence, indicating the inter-spin distance predicted by DFT. (**c**) Chemical structures of the two spin labels, with the principal axes of their zero-field splitting (ZFS) and g-tensors, respectively (red = x, green = y, blue = z). Key: EB (erythrosin B), Sar (sarcosine), Leu (L-leucine), Aib (α-aminoisobutyric acid), TOAC (2,2,6,6-tetramethylpiperidine-1-oxyl-4-amino-4-carboxylic acid), and Lol (L-leucinol).

The bis-labeled peptide **1** (Figure 1b) was chosen as a model compound for this study because of the rigid α-helical structure expected from its alternating Leu-Aib sequence. In vacuo DFT optimization of **1** supports the α-helical structure of the peptide backbone and predicts a distance of 1.9 nm between the two labels. Details on the synthesis and purification of **1** are given in the Materials and Methods section. A sarcosine linker was used instead of the more rigid direct attachment of the EB label to the N-terminus of the peptide via its carboxylate group in order to avoid the formation of the colorless spirolactam form [33]. This method is advantageous to previously reported strategies with similar chromophores, which involved the use of more expensive para-substituted derivatives of the chromophores as starting materials or introduced longer 5-atom linkers with additional unwanted conformational flexibility [22]. Our approach uses the more affordable non-derivatized form of EB and only introduces a 3-atom linker between the chromophore and the labeled molecule. Such an approach could also be expanded to other dyes, including EY and RB.

**Figure 2:**
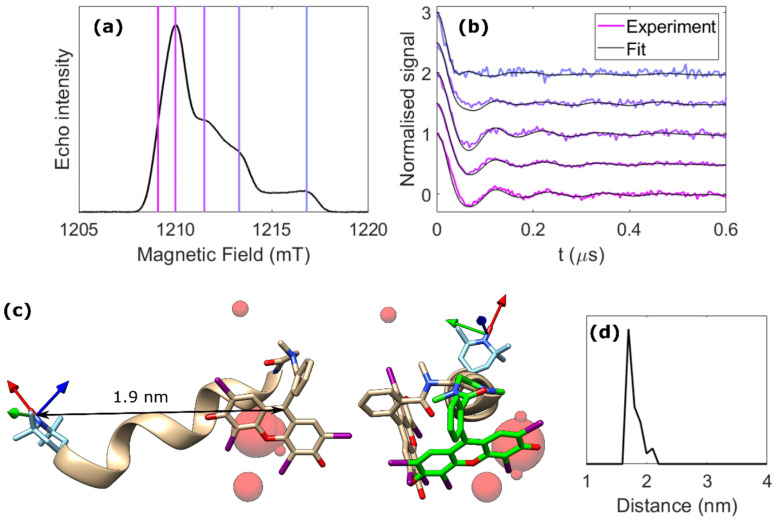
ReLaserIMD on the model peptide. (**a**) Echo-detected field-swept spectrum in the dark, showing the field positions where ReLaserIMD traces were acquired. (**b**) Background-corrected and modulation depth-normalized ReLaserIMD traces (colored lines) and corresponding orientationally dependent fits (black lines). Modulation depths before normalization were ~7% for all traces. (**c**) DFT-optimized structure of **1** (side and projection views) showing the different positions of the EB center determined by the fitting procedure as red spheres, relative to the nitroxide g-tensor frame (arrows: red = g_x_, green = g_y_, blue = g_z_). The diameter of the spheres is proportional to the number of times a single EB position contributes to the complete fit shown in panel b. The green structure on the right of the panel corresponds to another local energy minimum conformation identified by DFT. (**d**) Corresponding distance distribution.

The ReLaserIMD technique (Figure 1a) was applied at different field positions spanning the full width of the nitroxide EPR spectrum at Q-band (Figure 2a) to obtain an orientation-resolved set of dipolar traces. The orientation selection of the microwave detection pulses is evident from the appearance of a faster dipolar frequency component in the oscillating traces as the external magnetic field is increased (Figure 2b, colored lines). ReLaserIMD was chosen over Hahn-echo LaserIMD because of its more accurate determination of the experimental zero time [19], which is critical for the correct interpretation of the fast frequency components arising from orientational effects.

Orientation-dependent simulations were carried out using a previously published algorithm [8] and were used to fit the experimental dataset in an iterative least-squares global fitting process [35]. An initial fit using a molecular model generated around the DFT-optimized geometry of **1** was performed considering the photoexcited triplet spin density to be concentrated at the center of the EB moiety (Figure S9). This is a reasonable approximation as the light used here is not polarized and, consequently, there is no experimental photoselection for different orientations of the EB moiety with respect to the external magnetic field. The spread of the spin density over the EB moiety is included implicitly as the relative separation between EB spin density positions on consecutive simulated conformers was smaller than the size of the EB moiety and therefore several fitted points of the spin density could originate from the same molecular conformation of EB. Modulation depths were normalized to 1 for ease of analysis.

The results of this initial fit, in which the delocalization of the EB triplet spin density was not explicitly considered (Figure S9), were used to refine the model and a second fit was carried out including the delocalized EB triplet spin density calculated by DFT (Figure S8). In this case, the 20 best-fitting dipolar vectors linking the nitroxide spin density to the center of the EB moiety were used. The relative orientation between the g-frame of the nitroxide radical, which was fixed with respect to the dipolar vector for each chosen dipolar vector, and the zero-field splitting (ZFS) frame of the EB triplet were varied with three Euler angles. The simulation results show small variations in the calculated traces (Figure S9), which mainly arise due to the changing distances of each point of the spin density relative to the nitroxide as the EB orientation is varied for each dipolar vector. The results of this fit (Figure 2b, black lines) are in excellent agreement with the experimental data and the corresponding spin–spin distance distribution has the maximum at 1.8 nm (Figure 2d).

The conformational distribution of the molecule derived from the orientational analysis is plotted as spheres positioned at the center of the EB moiety with respect to the nitroxide g-frame, where the diameter of each sphere is proportional to the population of this particular conformation (Figure 2c). These results suggest the chromophore folds back onto the peptide backbone as predicted by DFT. However, the direction of this fold is different from the DFT structure with the minimum calculated energy (Figure 2c, beige structure). The flexibility in the sarcosine linker and at the N-terminus of the alpha helix means that other DFT-optimized structures with similar but slightly higher energies can be converged, leading to other local energy minima. Specifically, rotation around the C–N bond of the sarcosine linker yields a structure where the EB chromophore is closer to the results of the orientational analysis (Figure 2c, green structure). The fit results also show reasonable agreement of the *z*-axis of the EB ZFS-frame (concurrent with the long axis of the EB moiety) with the green structure (Figure S13). This provides further evidence that this structure may be the dominant conformation in frozen solution. In addition, it should be considered that all DFT calculations in this work were performed in vacuo. Consequently, the presence of solvent could stabilize alternative conformations of the peptide that involve further rotation around the sarcosine C–N bond and/or change the hydrogen bonding network at the N-terminus. 

A model-free analysis, involving a more time-consuming exploration of the full conformational space, rendered very similar results, with only a slightly wider conformational spread due to the unrestricted nature of this approach (Figures S14 and S16). These results validate the model-based analysis and confirm that the use of the minimum-energy DFT structure as a guide to the conformation space in which the model is constructed does not skew the conformational information obtained.

**Figure 3:**
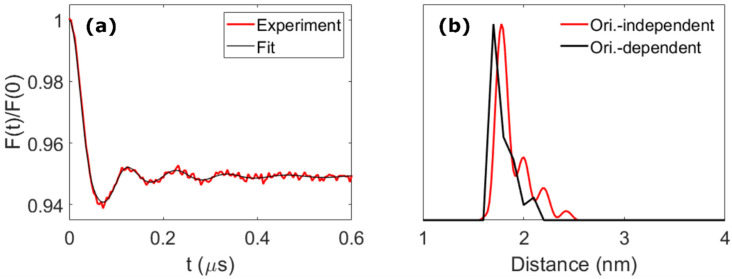
Orientational averaging. (**a**) Averaged form factor (red) and fit by Tikhonov regularization using *DeerAnalysis2019* (black). (**b**) Corresponding distance distribution (red) and distribution obtained from the orientation-dependent analysis (black, Figure 2d).

If only the spin–spin distance distribution is of interest, the dipolar traces measured across the nitroxide spectrum can be added together to average and thus remove the orientational effects, resulting in an orientation-independent form factor. This can be analyzed by Tikhonov regularization using standard software such as *DeerAnalysis* [36] (Figure 3a). For this system, the resulting distance distribution agrees well with that obtained from the orientation-dependent analysis and with the DFT prediction (Figure 3b), and the small deviations are likely a result of incomplete orientation averaging caused by the discreet nature of the traces measured. Comparison to an orientation-independent analysis of each individual dipolar trace by Tikhonov regularization shows that the traces acquired around the nitroxide spectral maximum are not subjected to strong orientational effects in the studied molecule and render very similar distance distributions (Figure S17). It is therefore possible, for this particular molecule, to measure a single dipolar trace at the spectral maximum and to analyze it without taking orientational effects into consideration. However, this is not a general result, and it might not be true for other bis-labeled molecules, as the orientational effects on the dipolar traces depend on the relative orientation between the nitroxide g-frame and the dipolar vector. If this relative orientation is not known a priori, orientation-dependent analysis or orientational averaging, involving the measurement of several dipolar traces at different parts of the spectrum of the detection spin center, are the recommended approaches.

**Figure 4:**
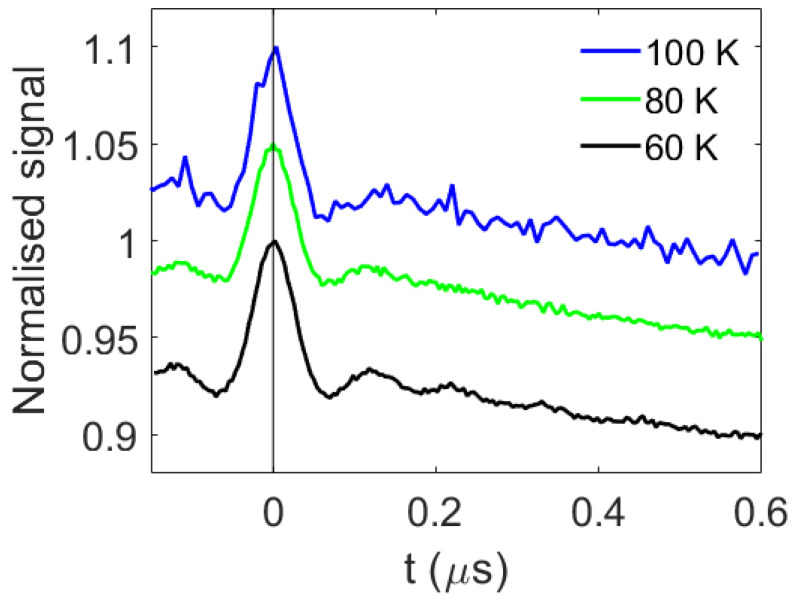
Raw ReLaserIMD traces acquired at the nitroxide signal maximum, at different temperatures: 60 (black), 80 (green), and 100 K (blue). The traces were measured for the same number of scans (750). The MNRs are 70, 73, and 23, respectively.

The ReLaserIMD measurements were repeated at temperatures up to 100 K (Figure 4 and Figure S19), demonstrating that LiPDS experiments can be carried out in the same conditions as conventional nitroxide–nitroxide PDS, without the need for expensive liquid helium or cryogen-free cryostats. This is a big step towards the widespread use of LiPDS in structural biology.

The modulation-to-noise ratio (MNR) of the traces, calculated as the modulation depth relative to the noise level at the end of the trace, is significantly reduced at 100 K; however, the values for the data measured at 60 and 80 K are very similar: 70 and 73, respectively (Figure 4 and Figure S19). This can be rationalized as the T_m_ times of the nitroxide measured at 60 and 80 K are very similar, whereas that measured at 100 K is approximately 3 times shorter (Figure S18 and Table S1).

Comparison of the data measured at 60 and 80 K shows that fewer oscillations are resolved in the trace recorded at 80 K compared to that recorded at 60 K (Figure 4 and Figure S19). One possible explanation for this could be an increase in the longitudinal spin relaxation and thermalization of the non-Boltzmann sublevel populations of the EB triplet at higher temperatures such that the spin state of EB generated by the laser pulse changes during the precession of the nitroxide spin, causing additional fluctuations in the nitroxide electron spin-echo intensity. It was not possible to observe a spin echo from the EB triplet at any of the temperatures studied, consequently it was not possible to measure an inversion-recovery or echo-detected delay after the flash experiment to determine the relaxation of the EB center and investigate this hypothesis. An alternative explanation is that, as the temperature increases, molecular motions also increase, and these motions may broaden the distribution of the dipolar frequencies observed at higher temperatures, leading to faster damping of the oscillations [37,38]. 

## 3. Materials and Methods

### 3.1. Sample Preparation

The peptide sequence in **1** was obtained by solid-phase peptide synthesis on a 2-chlorotrityl resin preloaded with Lol. The sequence contains several Aib residues known to induce helical conformations. The synthetic procedure followed for this new TOAC-containing peptide resembles those previously described [39,40]. All reagents and solvents were purchased from either Merck KGaA (Darmstadt, Germany) or Iris Biotech GmbH (Marktredwitz, Germany). Since Aib is a poorly reactive residue, all coupling steps were based on Oxyma Pure and N,N’-diisopropylcarbodiimide activation and were performed twice. EB was linked to the N-terminal Sar residue under the same experimental conditions, but the coupling reaction was repeated three times. The peptide was cleaved from the resin by repeated treatments with 30% 1,1,1,3,3,3-hexafluoroisopropanol in dichloromethane, purified by preparative reversed-phase (RP)-HPLC on a Phenomenex C4 column (40 × 250 mm, 10 µ, 300 Å) using a Pharmacia (GE healthcare, US) system (flow rate 10 mL min^−1^, λ = 206 nm). Eluant A, H_2_O/CH_3_CN 9:1 *v*/*v*; Eluant B, CH_3_CN/H_2_O 9:1 *v*/*v*; gradient 75–100–100% B in 12 + 10 min. The purified fractions were characterized by analytical RP-HPLC on a Phenomenex C4 column (4.6 × 250 mm, 5 µ, 300 Å) using the same Pharmacia system. Eluant A, H_2_O/CH_3_CN 9:1 *v*/*v* + 0.05% trifluoroacetic acid (TFA); Eluant B, CH_3_CN/H_2_O 9:1 *v*/*v* + 0.05% TFA. High-resolution electrospray ionization mass spectrometry (ESI-HRMS) was performed on a Waters Micromass Xevo instrument (Milford, MA, USA) in negative mode: [M-H]^-^ found = 2107.5442, [M-H]^-^ calcd. = 2107.5584. Analytical HPLC and MS indicate that the peptide was obtained at good purity, with a yield after purification of 9% (Figures S1 and S2).

Samples for UV-Vis absorption spectroscopy were prepared to 1 µM of **1** in ethanol (>95 %, Merck, Gillingham, UK).

Samples for EPR were prepared to 50 µM of **1** for Q-band and 100 µM of EB (95 %, Aldrich) for X-band in ethanol-d_6_ (anhydrous, >99.5 atom %, Merck, Gillingham, UK). Samples were degassed by several freeze-pump-thaw cycles, sealed inside quartz tubes (3 mm outer diameter for Q-band, 4 mm for X-band), and flash-frozen in liquid nitrogen prior to insertion into the spectrometer.

### 3.2. Spectroscopy

UV-Vis absorption spectra were acquired in 10 mm quartz cuvettes using a UV-Vis spectrophotometer (Cary 60, Agilent, Santa Clara, CA, USA). Transient absorption spectra were acquired in sealed 2 mm glass cells using a nanosecond transient absorption spectrometer (EOS, Ultrafast systems, Sarasota, FL, USA) after photoexcitation by a Nd:YAG-pumped optical parametric generator (PL2210 and PG403, Ekspla, Vilnius, Lithuania).

Pulsed EPR experiments were carried out in an ElexSys E580 spectrometer (Bruker, Billerica, MA, USA), using an ER 5106 QT2 resonator (Bruker, Billerica, MA, USA) at Q-band (34 GHz). The temperature was maintained at 60 K using liquid helium or at 80, 100, and 120 K using liquid nitrogen, in a CF935 cryostat (Oxford Instruments, Abingdon, UK) with an ITC103 temperature controller (Oxford Instruments, Abingdon, UK). Laser excitation was provided by an OPO (Opolette355, Opotek, Carlsbad, CA, USA) operated at a repetition rate of 20 Hz (5 ns pulses) at a wavelength of 532 nm, with an energy of 2 mJ per pulse. The wavelength was chosen to be close to the absorption maximum of the sample (Figure S3). The beam was passed through a depolarizer (Thorlabs, Newton, NJ, USA) before being directed into the spectrometer.

Rectangular microwave pulses of π = 40 ns and π/2 = 20 ns were used for all pulsed EPR experiments. Field sweep and phase-memory time experiments were performed using a standard Hahn echo sequence (π/2 – τ – π – τ – echo). For the inversion-recovery experiments, this sequence was preceded by an inversion pulse (π – T – π/2 – τ – π – τ – echo). ReLaserIMD used the refocused echo three-pulse sequence shown in Figure 1a: π/2 – τ_1_ – π – τ’ – laser – τ’’ – π – τ_2_ – echo, with τ_1_ = 600 ns, τ_2_ = 200 ns, and τ_1_ + τ_2_ = τ’ + τ’’. The experiment was carried out at 5 different values of the external magnetic field (c.a. 1209.1, 1210.0, 1211.5, 1213.3, and 1216.8 mT) resonant with different parts of the nitroxide spectrum in order to probe orientation selection. The raw dipolar traces were phase- and background-corrected to obtain orientation-dependent ReLaserIMD form factors. For the orientation-independent analysis, these form factors were averaged weighted by the corresponding spectral intensities of the nitroxide to obtain an orientation-independent form factor, which was then analyzed via Fourier transform and Tikhonov regularization using the MATLAB^®^ *DeerAnalysis2019* [36] routine to extract a distance distribution. MNRs were calculated using the modulation depth of the ReLaserIMD traces and noise intensities estimated as the root-mean-square deviation (RMSD) of the form factors after complete damping of the dipolar oscillations.

The EPR characterization of free EB was carried out in an ElexSys E680 spectrometer (Bruker, Billerica, MA, USA) using an EN 4118X-MD5 resonator (Bruker, Billerica, MA, USA) at X-band (9.7 GHz) and 20 K, with the same laser settings reported above. For the variable delay-after-flash (DAF) measurement (laser – DAF – π/2 – τ – π – τ – echo), the resonator was over-coupled. For the time-resolved EPR (trEPR) measurement, the resonator was critically coupled, and no field modulation or phase-sensitive detection was used. The signal was averaged between 0.5 and 2.0 ns after the laser flash, around the signal maximum of the time trace.

### 3.3. DFT Calculations

Initial geometries for molecule **1** were built in UCSF Chimera [41]. All DFT calculations were performed in vacuo. Geometry optimizations of **1** in the ground state were carried out using Gaussian^®^ 16 (revision A.03) [42], with the functional PBE1PBE, the basis set 6–31 g (d), and a spin multiplicity of 2. Iodine atoms in EB were replaced by hydrogens to speed up the calculation. The spin density in the nitroxide radical was obtained by single-point calculation using the functional BP86 and the basis set Def2-SVP.

The geometry optimization and spin density calculation of EB in the triplet state (spin multiplicity of 3) was carried out in Orca (release version 4.2.0) [43], using the functional BP86 and the basis set Def2-TZVP. Only the EB-Sar-Leu segment of the peptide was included. The resolution of identity (RI) approximation, with the auxiliary basis set def2/J, was used.

### 3.4. Orientation-Dependent Simulations

The protocol for orientation-dependent simulations was first described for conventional PDS by Lovett et al. [8] and adapted for LiPDS by Bowen et al. [30]. ReLaserIMD simulations were carried out in the g-tensor frame of the nitroxide radical (Figure 1b). The ‘pump pulse’ used in the simulation was considered to excite all orientations of EB with respect to the external magnetic field, reflecting the fact that the laser light used in this experiment was depolarized. The spin Hamiltonian parameters used to simulate the EPR spectra of the nitroxide radical and EB triplet were obtained from fitting the echo-detected field-swept (Figure 2a) and trEPR (Figure S4) spectra, respectively, using the MATLAB^®^-based *EasySpin* routine (*pepper* function) [44]. Nitroxide radical: g = [2.0086, 2.0052, 2.0014], g-strain = [0.0011, 0.0020, 0.0011], A_N_ = [2.17, 13.8, 103.2] MHz and A-strain = [0, 0, 1] mT. EB triplet: D = 3486 MHz, E = 328 MHz, D-strain = 990 MHz, E-strain = 0 MHz, Gaussian linewidth = 3 mT, *p_x_* = 0.6, *p_y_* = 0.4, *p_z_* = 0.0.

A library of pre-simulated traces was fitted to the experimental data following the protocol similar to that reported by Marko et al. [35]. For the model-based fit, an initial model consisting of a cone of 1200 dipolar vectors around the DFT-optimized geometry was generated (Δθ=30°, Δr=0.2 nm) and the corresponding dipolar traces at the 5 different field positions were simulated considering the spin density of the EB triplet to be concentrated at a single point in space in the center of the chromophore. The 20 dipolar vectors found to give the largest contributions to this first fit were then selected to generate a second model, where the spin delocalization of the EB and orientation of the EB moiety with respect to the nitroxide were considered. The orientation of the EB moiety with respect to the nitroxide was varied by Δα=Δβ=Δγ= 45° in steps of 22.5°, where α,β,γ are the Euler angles defining the orientation of the ZFS-tensor frame of the EB triplet with respect to the g-tensor frame of the nitroxide radical. The electron spin delocalization in the EB triplet state was included as calculated by DFT. For the model-free fit, one quarter of a spherical shell of Δr= 0.5 nm around r= 1.8 nm, containing 5115 dipolar vectors, was used to simulate the library of dipolar traces, again considering the spin density of the EB triplet to be concentrated at the center of the chromophore. A total of 50 least-squares fitting iterations was sufficient to reach convergence of RMSD from the experimental data in all the cases (Figures S10, S12 and S15).

## 4. Conclusions

The variety of choice in photoswitchable spin labels and the affordability of the experiments are critical for the expansion of light-induced pulsed electron paramagnetic resonance dipolar spectroscopy (LiPDS) to become a widespread and complementary methodology to conventional PDS. With this work, we have taken an important step in this direction by introducing a new photoswitchable spin label and by removing the need for expensive cryogenics in these experiments.

We reported, for the first time, the use of the photoexcited triplet state of erythrosin B (EB) as a photoswitchable spin label for LiPDS. Its strong light absorption in the green and its high triplet quantum yield allow for low-demanding photoexcitation using the second harmonic of a simple Nd:YAG laser. In addition, its small size, biocompatibility, and commercial availability in functionalized forms for biolabeling make it an ideal candidate for biological applications.

Employing the refocused laser-induced magnetic dipole (ReLaserIMD) technique to measure the dipolar interaction between the EB triplet and a permanent nitroxide radical in a rigid model peptide, we exploited the orientational effects in the dipolar traces to obtain information on both the distance and relative orientation between the two spin-bearing moieties. The agreement between different data analysis approaches and density-functional theory calculations demonstrates the robustness of this methodology. With this, we showed the importance of capturing the orientational effects underlying these experiments, which can then be correctly averaged to perform an orientation-independent analysis free of orientational artefacts if only the distance distribution is of interest. 

In addition, we demonstrated the feasibility of these experiments above liquid nitrogen temperatures, removing the need for expensive liquid helium or cryogen-free cryostats, which often restricts the accessibility of LiPDS to researchers.

## Data Availability

Data is available from the authors on request and will be deposited in a data repository.

## Supplementary Materials

The following supporting information can be downloaded at: https://www.mdpi.com/article/10.3390/molecules27217526/s1, Figure S1. Analytical HPLC of **1** after purification. Experimental conditions: Phenomenex C4 column (4.6 × 250 mm, 5 µ, 300 Å). Eluant A, H_2_O/CH_3_CN 9:1 *v*/*v* + 0.05% trifluoroacetic acid (TFA); Eluant B, CH_3_CN/H_2_O 9:1 *v*/*v* + 0.05% TFA. Gradient: 75–100%B in 7 min., Figure S2. ESI-HRMS spectrum of **1** after purification, acquired in negative mode, showing the most informative regions. [M-H]^-^ found = 2107.5442, [M-H]^-^ calcd. = 2107.5584, Figure S3. Room-temperature UV-Vis absorption spectrum of **1** in ethanol, Figure S4. X-band trEPR spectrum of EB measured after photoexcitation at 532 nm, 20 K (black) and simulation (red) using *EasySpin* [44], *pepper* function, with the following triplet state spin Hamiltonian parameters: D = 3486 MHz, E = 328 MHz, D-strain = 990 MHz, E-strain = 0 MHz, Gaussian linewidth = 3 mT, *p_x_* = 0.6, *p_y_* = 0.4, *p_z_* = 0.0, Figure S5. Characterization of the relaxation of the nitroxide spectral maximum in the dark, 60 K. (a) Inversion-recovery experiment (red) with biexponential fit (black) rendering lifetimes of (0.315 ± 0.008) and (1.03 ± 0.04) ms, with relative weights of 0.73:0.27. The signal has been plotted as a positive decay for ease of analysis. (b) Phase-memory-time experiment (red) with mono-exponential fit rendering a lifetime of (1.07 ± 0.01) µs (black), Figure S6. Transient absorption spectroscopy of free EB in ethanol at 100 K. (a) Absorption spectrum of the EB triplet formed by photoexcitation at 532 nm, time-averaged around the signal maximum. (b) Time decay of the triplet absorption at 600 nm (circles) and mono-exponential fit (red line) with a lifetime of 0.69 ms, Figure S7. Variable delay-after-flash (DAF) spin-echo experiment with free EB in d_6_-ethanol at 20 K, with photoexcitation at 532 nm. Echo intensity time trace measured at the most intense feature of the EB triplet EPR spectrum (red) and biexponential fit with lifetimes of 0.01 and 0.1 ms and relative weights of 0.26:0.74, Figure S8. Calculated electronic spin densities for the two labels (EB triplet, left; nitroxide radical, right) used in the orientation-dependent analysis. See the Materials and Methods section for the computational details, Figure S9. Results of the model-based fit with the triplet spin density in the center of the EB moiety. (a) Echo-detected field-swept spectrum in the dark, showing the field positions where ReLaserIMD traces were acquired. (b) Background-corrected and modulation depth-normalized ReLaserIMD traces (thick lines) and corresponding orientationally dependent fits (thin lines). (c) DFT-optimized structure of **1** showing the different positions of the EB center determined by the fitting procedure as red spheres, relative to the nitroxide g-tensor frame (arrows: red = g_x_, green = g_y_, blue = g_z_). The diameter of the spheres is proportional to the number of times a single EB position contributes to the complete fit shown in panel b. (d) Corresponding distance distribution. Figure S10. RMSD form the fit in Figure S9, Figure S11. Simulated ReLaserIMD traces with the dipolar vector most contributing to the best fit in Figure S7, using the delocalized EB triplet spin density calculated by DFT and changing the orientation of the EB chromophore with the Euler angles α, β, and γ by 45° in steps of 22.5°. The colors correspond to the different values of the external magnetic field as introduced in Figure S9a. Figure S12. RMSD from the fit in Figure 2, Figure S13. Orientations of the D-tensor (zero-field splitting tensor) z-axis of the EB triplet (parallel to the long axis of the chromophore) for the dipolar vector most contributing to the best fit in Figure 2, Figure S14. Results of the model-free fit with the triplet spin density in the center of the EB moiety. (a) Echo-detected field-swept spectrum in the dark, showing the field positions where ReLaserIMD traces were acquired. (b) Background-corrected and modulation depth-normalized ReLaserIMD traces (thick lines) and corresponding orientationally-dependent fits (thin lines). (c) DFT-optimized structure of **1** showing the different positions of the EB center determined by the fitting procedure as red spheres, relative to the nitroxide g-tensor frame (arrows: red = g_x_, green = g_y_, blue = g_z_). The diameter of the spheres is proportional to the number of times a single EB position contributes to the complete fit shown in panel b. (d) Corresponding distance distribution, Figure S15. RMSD form the fit in Figure S13, Figure S16. Comparison between model-based (red, Figure S9) and model-free (blue, Figure S15) fits, both with single-point spin density, Figure S17. Orientation-independent analysis of individual dipolar traces. (a) Echo-detected field-swept spectrum in the dark, showing the field positions where ReLaserIMD traces were acquired. (b) Background-corrected and modulation depth-normalized ReLaserIMD traces (thick lines) and orientationally-independent fits by Tikhonov regularization using *DeerAnalysis2019* [36] (black). (c) Corresponding distance distributions, Figure S18. Characterization of the relaxation of the nitroxide spectral maximum in the dark at different temperatures: 60 K (black), 80 K (green), 100 K (blue), and 120 K (red). (a) Inversion-recovery experiments (thick lines) with biexponential fits (thin lines). The signal was plotted as a positive decay for ease of analysis. (b) Phase-memory-time (T_m_) experiments (thick lines) with mono-exponential fits (thin lines). The lifetimes determined from the fits are reported in Table S1, Figure S19. Background-corrected and modulation-depth-normalized ReLaserIMD traces acquired at the nitroxide signal maximum, at different temperatures: 60 K (black), 80 K (green), 100 K (blue), and 120 K (red). The traces have been averaged for the following number of scans: 1875, 2520, 6960, and 29,350, respectively. A time step of 8 ns was used to acquire the traces at 100 K and 120 K in order to reduce the acquisition time while a 4 ns step size was used at 60 K and 80 K. Modulation depths were ~7% before normalization in all cases, Table S1. Lifetimes extracted from the fits in Figure S18.

## Sample Availability

Samples of bis-labelled peptide **1** are available from the authors.
