# Supplementary material for: Erythrosin B as a New Photoswitchable Spin Label for Light-Induced Pulsed EPR Dipolar Spectroscopy
Source: Molecules. 2022 Nov 3;27(21):7526. doi: 10.3390/molecules27217526 (PMC9657417; doi:10.3390/molecules27217526)
Supplement: Supplementary file 1 [file molecules-27-07526-s001.zip › molecules-1924474-supplementary.pdf]

Supplementary Materials for

Erythrosin B as a New Photoswitchable Spin Label for Light-Induced Pulsed EPR Dipolar Spectroscopy

Arnau Bertran,[1] Laura Morbiato,[2] Sara Aquilia,[2] Laura Gabbatore,[2] Marta De Zotti,[2,3] Christiane R. Timmel,[1] Marilena Di Valentin[2,3,] and Alice M. Bowen[4,*]*

[1] Centre for Advanced Electron Spin Resonance and Inorganic Chemistry Laboratory, Department of Chemistry, University of Oxford, South Parks Road, Oxford OX1 3QR, United Kingdom.

[2] Department of Chemical Sciences, University of Padova, Via Marzolo 1, 35131 Padova, Italy.

[3] Centro Interdipartimentale di Ricerca "Centro Studi di Economia e Tecnica dell'energia Giorgio Levi Cases", 35131 Padova, Italy.

[4] The National Research Facility for Electron Paramagnetic Resonance, Department of Chemistry and Photon Science Institute, The University of Manchester, Oxford Road, Manchester M13 9PL, United Kingdom.

CONTENTS

S1. Synthesis

S2. Spectroscopic characterization

S3. Orientation-dependent analysis

S4. Orientation-independent analysis

S5. High-temperature experiments

REFERENCES

S1. Synthesis

UNICORN 5.11 (Build 407)
Result file: C:\...\default\Sara Aquilia\EB Leu Aib TOAC lol\preparativa 9 EBLUTOACLOL cuore picco a 23 min 50uL 75 100 WL 270 300 001

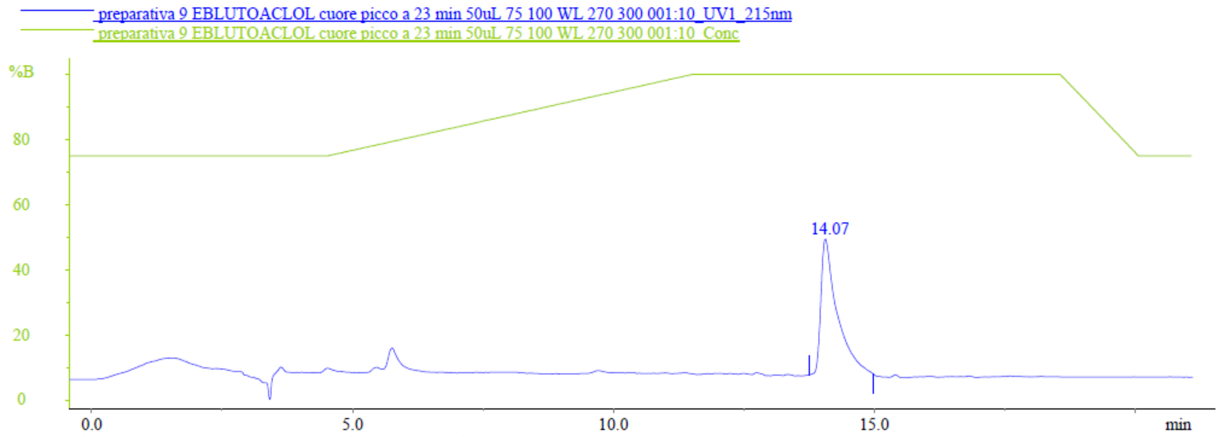


```
preparativa 9 EBLUTOACLOL cuore picco a 23 min 50uL 75 100 WL 270 300 001:10_UV1_215nm@01,PEAK1

No      Ret        Area    Area/Peak area     Height
        min     mAU*min          (time) %        mAU
 1    14.07     50.8365            100.00    135.683

     Total number of detected peaks = 201
     Total area = 118.6037  mAU*min
     Area in evaluated peaks = 50.8365  mAU*min
     Ratio peak area / total area = 0.428625
     Total peak width = 1.24 min
     Column height = 25.00 cm
     Calculated from : preparativa 9 EBLUTOACLOL cuore picco a 23 mi
     Baseline : preparativa 9 EBLUTOACLOL cuore picco a 23 min 50uL
     Peak rejection on:
       Maximum number of peaks: 20
```

Figure S1. Analytical HPLC of **1** after purification. Experimental conditions: Phenomenex C4 column (4.6 x 250 mm, 5 µ, 300 Å). Eluant A, H_2O/CH_3CN 9:1 v/v + 0.05% trifluoroacetic acid (TFA); Eluant B, CH_3CN/H_2O 9:1 v/v + 0.05% TFA. Gradient: 75-100%B in 7 min.

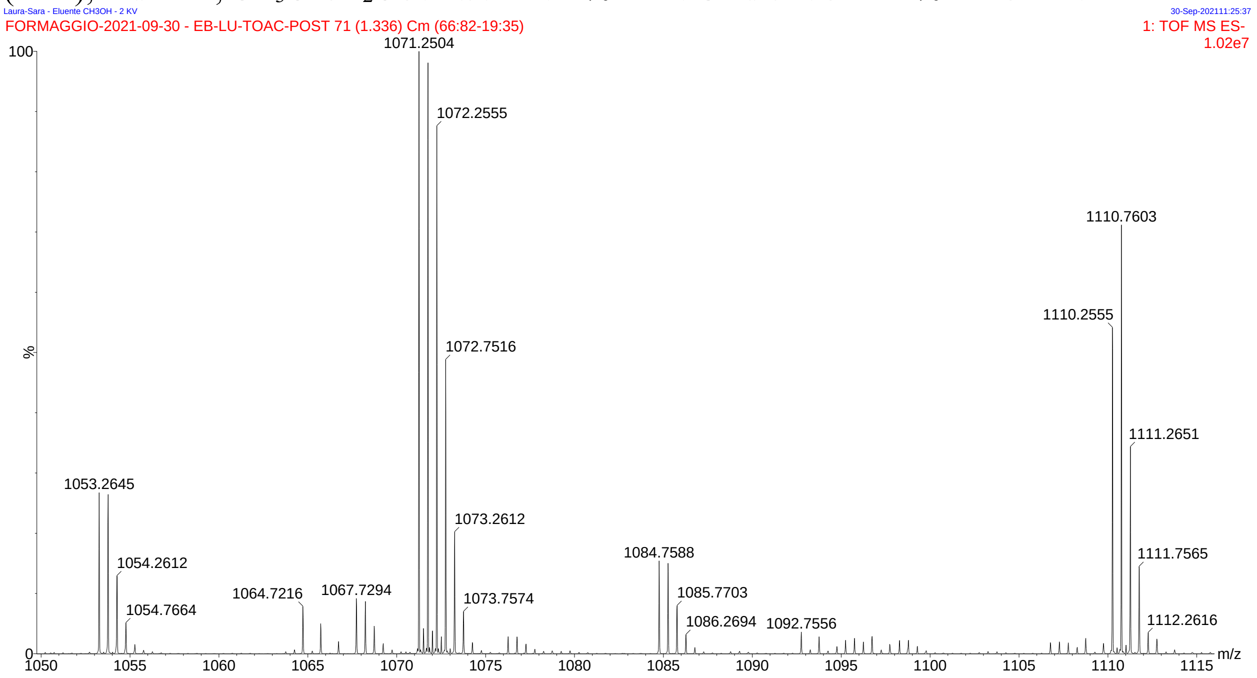

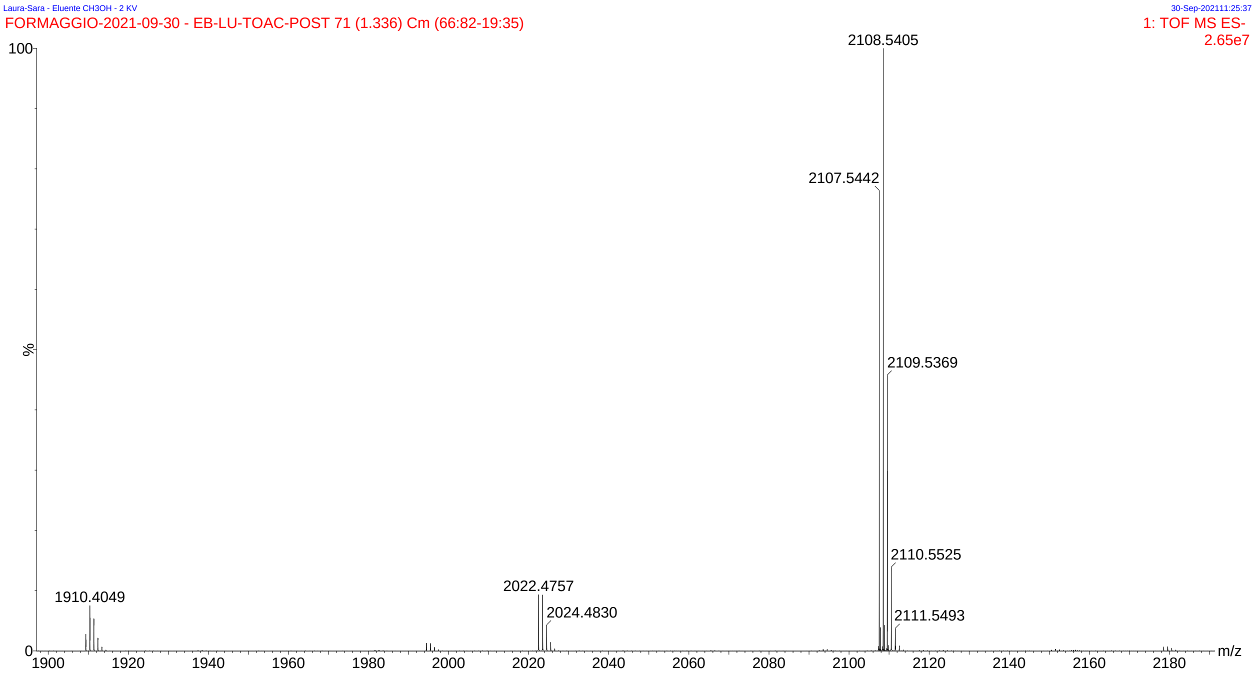

Figure S2. ESI-HRMS spectrum of **1** after purification, acquired in negative mode, showing the most informative regions. $[M-H]^-$ found = 2107.5442, $[M-H]^-$ calcd. = 2107.5584.

S2. Spectroscopic characterization

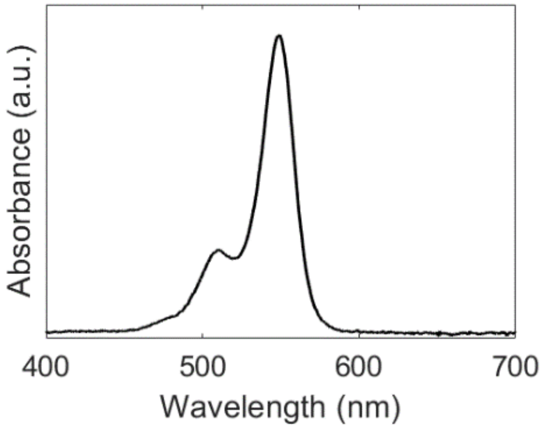

Figure S3. Room-temperature UV-Vis absorption spectrum of **1** in ethanol.

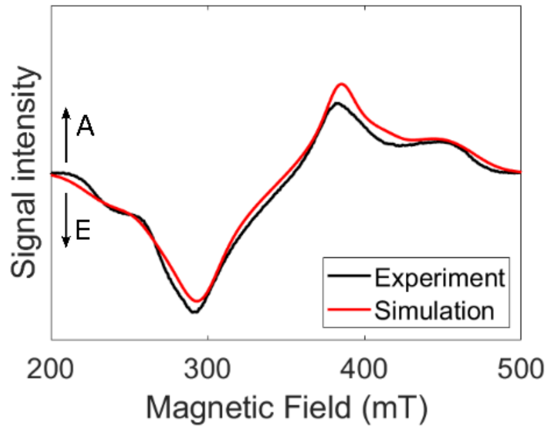

Figure S4. X-band trEPR spectrum of EB measured after photoexcitation at 532 nm, 20 K (black) and simulation (red) using *EasySpin* [1], *pepper* function, with the following triplet state spin Hamiltonian parameters: D = 3486 MHz, E = 328 MHz, D-strain = 990 MHz, E-strain = 0 MHz, Gaussian linewidth = 3 mT, p_x = 0.6, p_y = 0.4, p_z = 0.0.

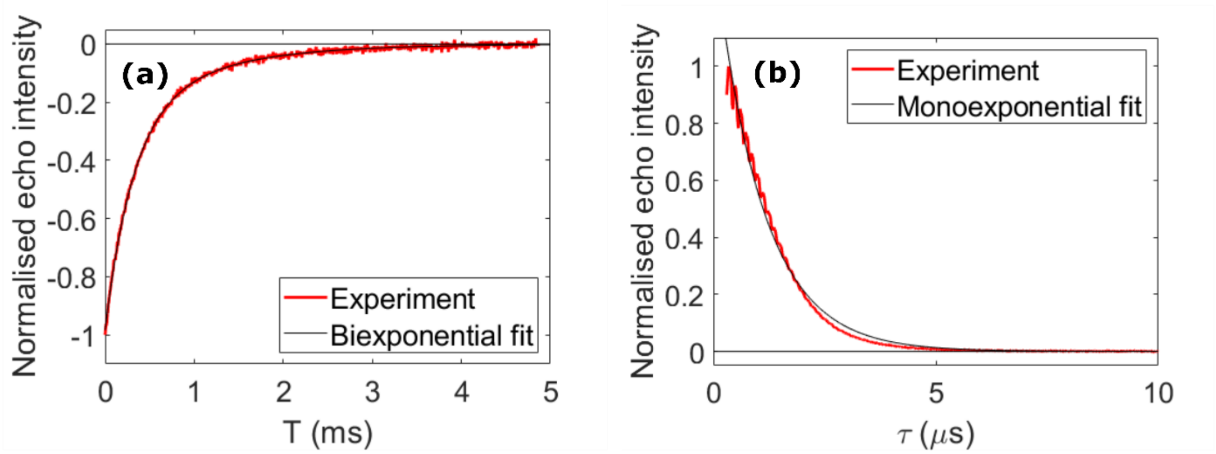

Figure S5. Characterization of the relaxation of the nitroxide spectral maximum in the dark, 60 K. (a) Inversion-recovery experiment (red) with biexponential fit (black) rendering lifetimes of (0.315 ± 0.008) and (1.03 ± 0.04) ms, with relative weights of 0.73 : 0.27. The signal has been plotted as a positive decay for ease of analysis. (b) Phase-memory-time experiment (red) with mono-exponential fit rendering a lifetime of (1.07 ± 0.01) μs (black).

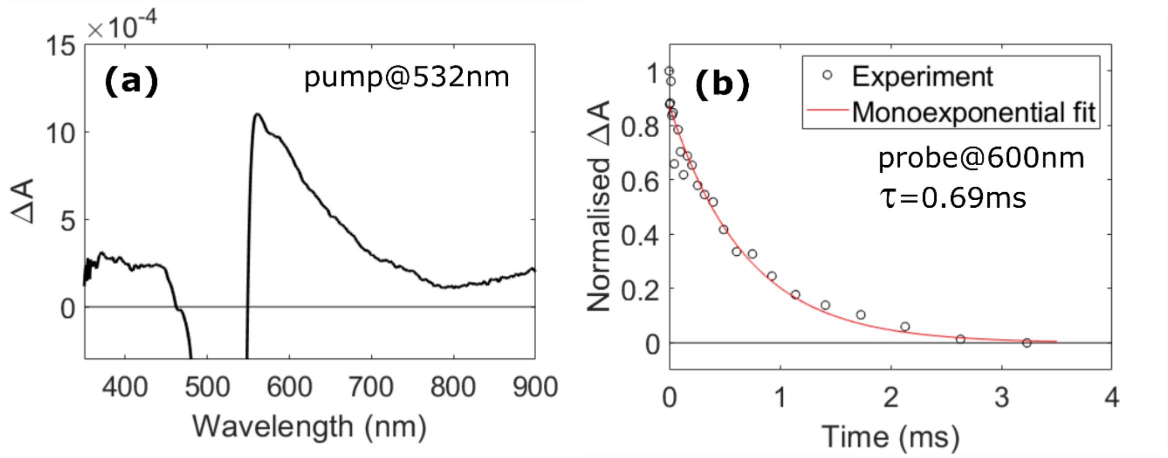

Figure S6. Transient absorption spectroscopy of free EB in ethanol at 100 K. (a) Absorption spectrum of the EB triplet formed by photoexcitation at 532 nm, time-averaged around the signal maximum. (b) Time decay of the triplet absorption at 600 nm (circles) and mono-exponential fit (red line) with a lifetime of 0.69 ms.

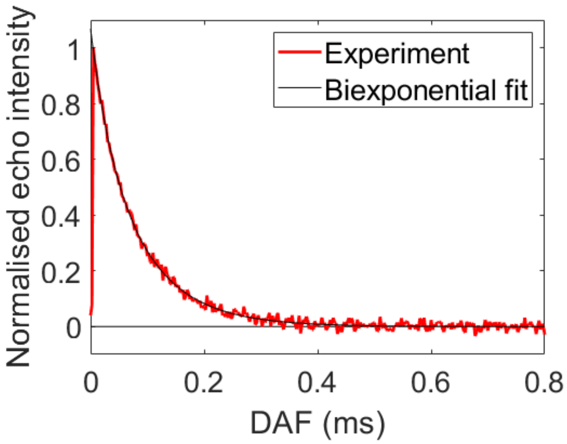

Figure S7. Variable delay-after-flash (DAF) spin-echo experiment with free EB in d_6-ethanol at 20 K, with photoexcitation at 532 nm. Echo intensity time trace measured at the most intense feature of the EB triplet EPR spectrum (red) and biexponential fit with lifetimes of 0.01 and 0.1 ms and relative weights of 0.26 : 0.74.

S3. Orientation-dependent analysis

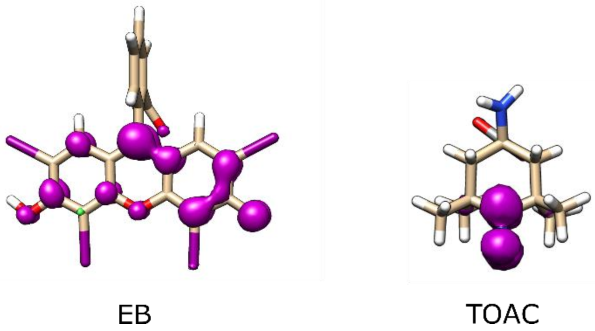

Figure S8. Calculated electronic spin densities for the two labels (EB triplet, left; nitroxide radical, right) used in the orientation-dependent analysis. See Materials and Methods section for the computational details.

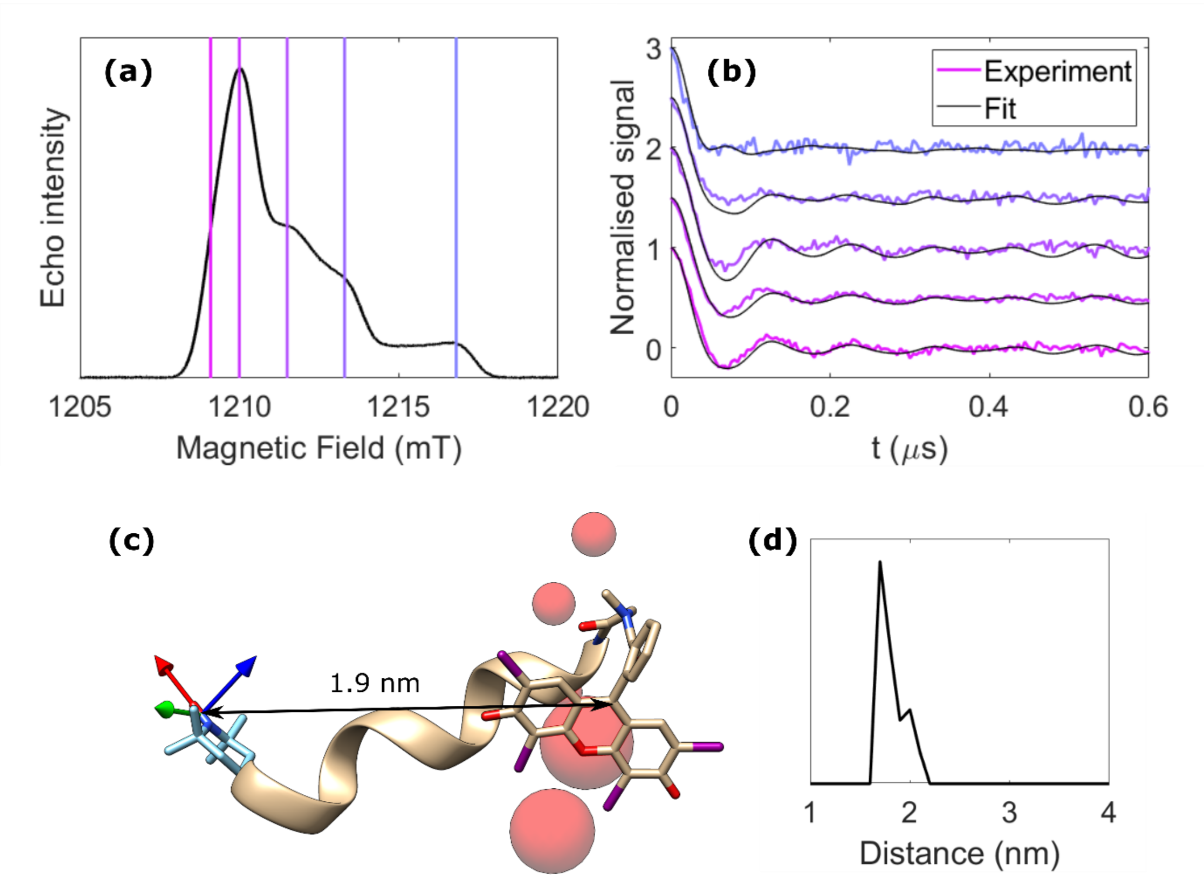

Figure S9. Results of the model-based fit with the triplet spin density in the center of the EB moiety. (a) Echo-detected field-swept spectrum in the dark, showing the field positions where ReLaserIMD traces were acquired. (b) Background-corrected and modulation depth-normalized ReLaserIMD traces (thick lines) and corresponding orientationally-dependent fits (thin lines). (c) DFT-optimized structure of **1** showing the different positions of the EB center determined by the fitting procedure as red spheres, relative to the nitroxide g-tensor frame (arrows: red = g_x, green = g_y, blue = g_z). The diameter of the spheres is proportional to the number of times a single EB position contributes to the complete fit shown in panel b. (d) Corresponding distance distribution.

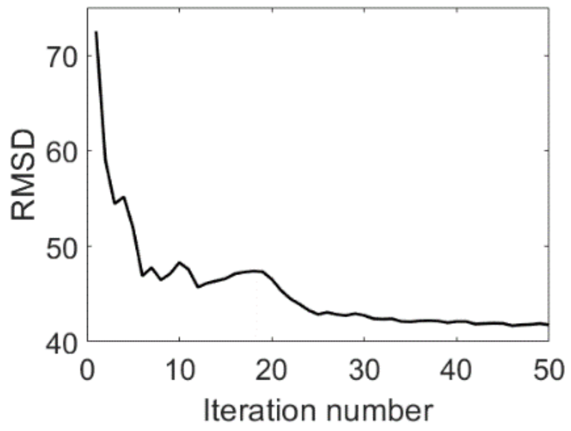

Figure S10. RMSD form the fit in Figure S9.

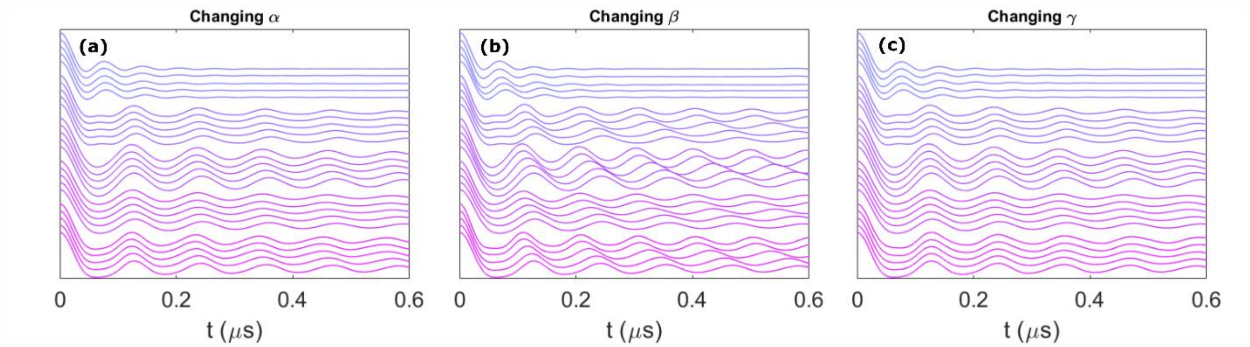

Figure S11. Simulated ReLaserIMD traces with the dipolar vector most contributing to the best fit in Figure S9, using the delocalized EB triplet spin density calculated by DFT and changing the orientation of the EB chromophore with the Euler angles α, β and γ by 45° in steps of 22.5°. The colors correspond to the different values of external magnetic field as introduced in Figure S9 (a).

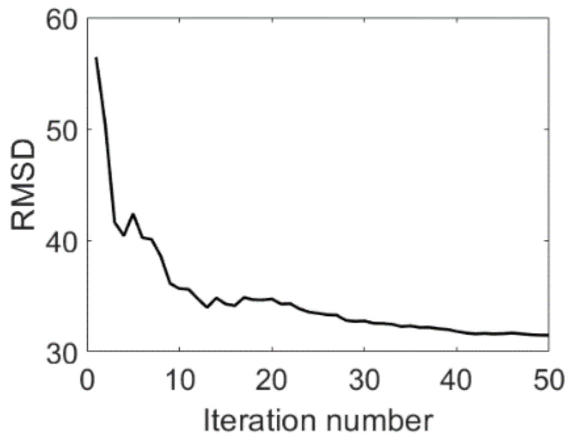

Figure S12. RMSD from the fit in Figure 2.

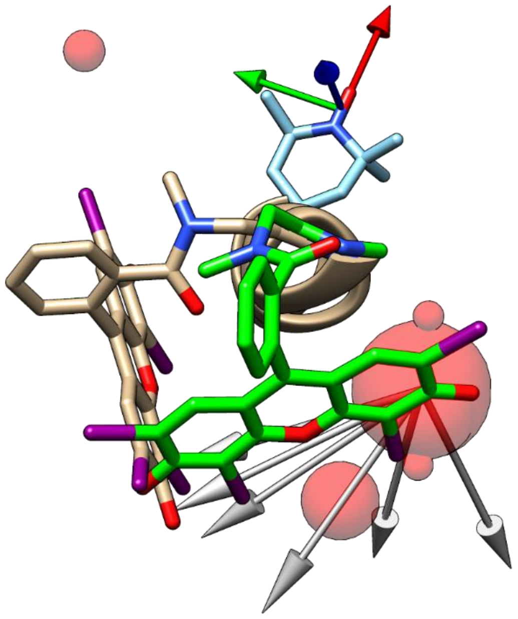

Figure S13. Orientations of the D-tensor (zero-field splitting tensor) z-axis of the EB triplet (parallel to the long axis of the chromophore) for the dipolar vector most contributing to the best fit in Figure 2.

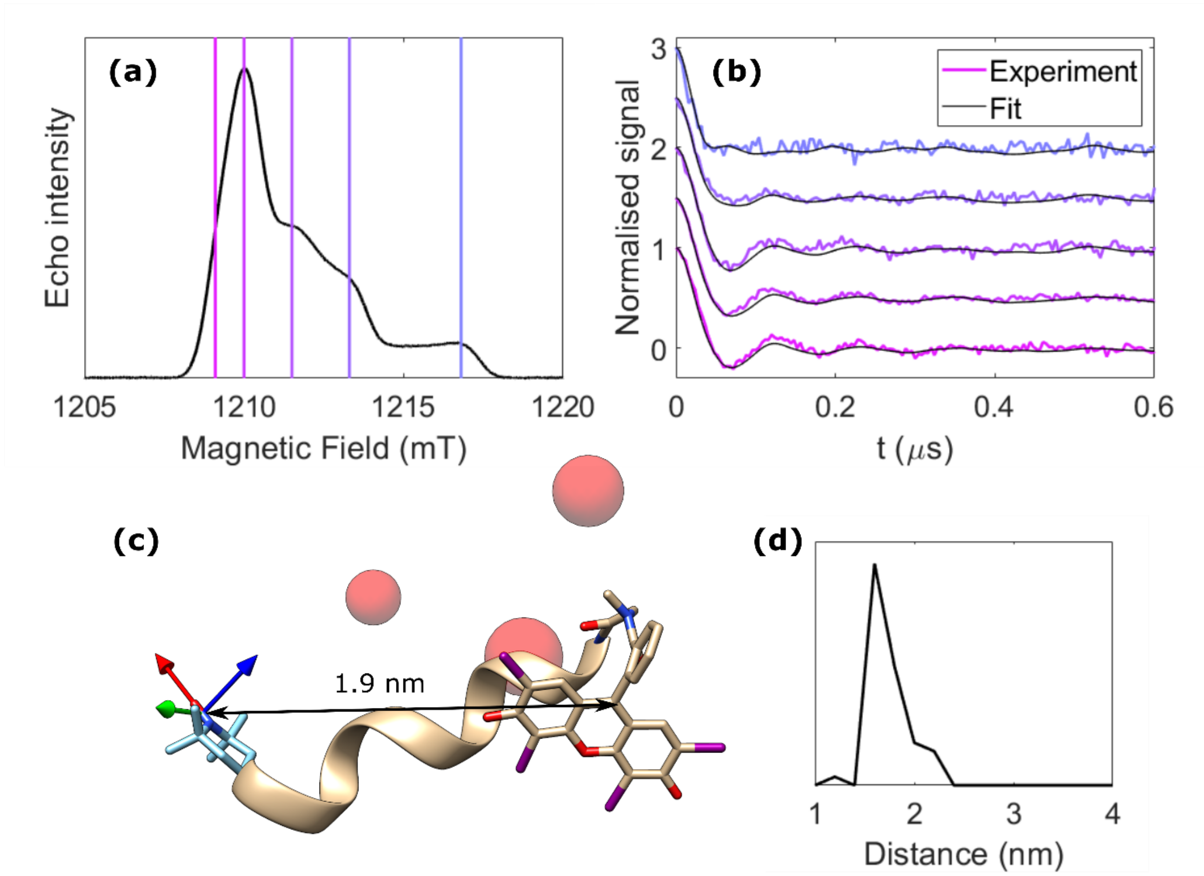

Figure S14. Results of the model-free fit with the triplet spin density in the center of the EB moiety. (a) Echo-detected field-swept spectrum in the dark, showing the field positions where ReLaserIMD traces were acquired. (b) Background-corrected and modulation depth-normalized ReLaserIMD traces (thick lines) and corresponding orientationally-dependent fits (thin lines). (c) DFT-optimized structure of **1** showing the different positions of the EB center determined by the fitting procedure as red spheres, relative to the nitroxide g-tensor frame (arrows: red = g_x, green = g_y, blue = g_z). The diameter of the spheres is proportional to the number of times a single EB position contributes to the complete fit shown in panel b. (d) Corresponding distance distribution.

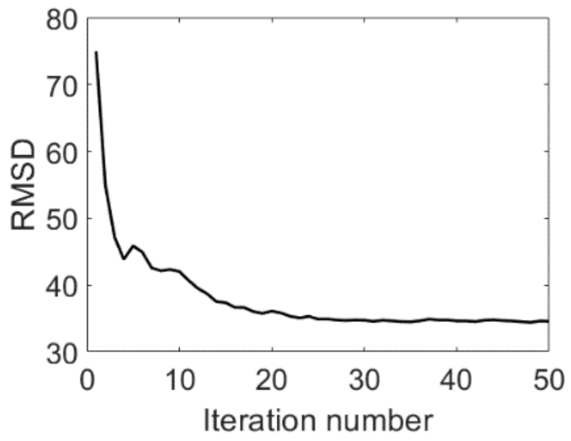

Figure S15. RMSD form the fit in Figure S13.

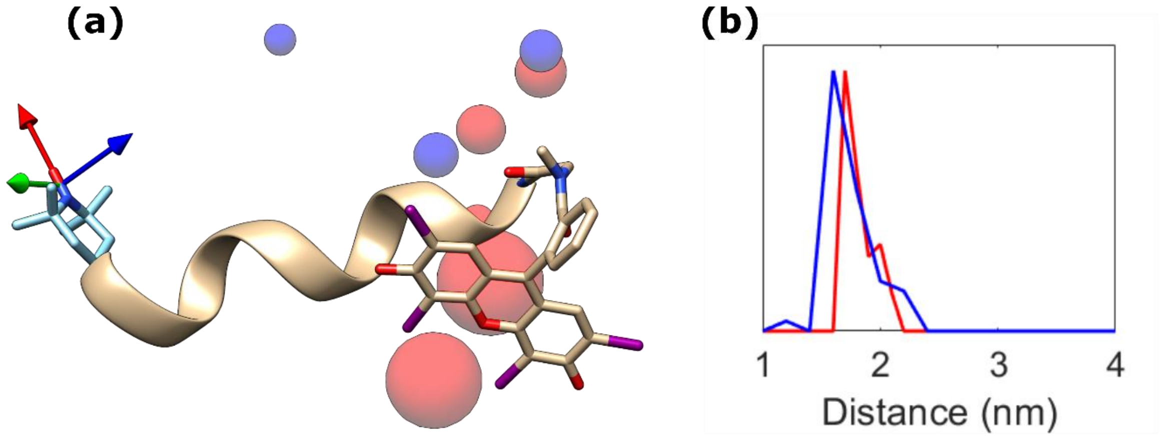

Figure S16. Comparison between model-based (red, Figure S9) and model-free (blue, Figure S15) fits, both with single-point spin density.

S4. Orientation-independent analysis

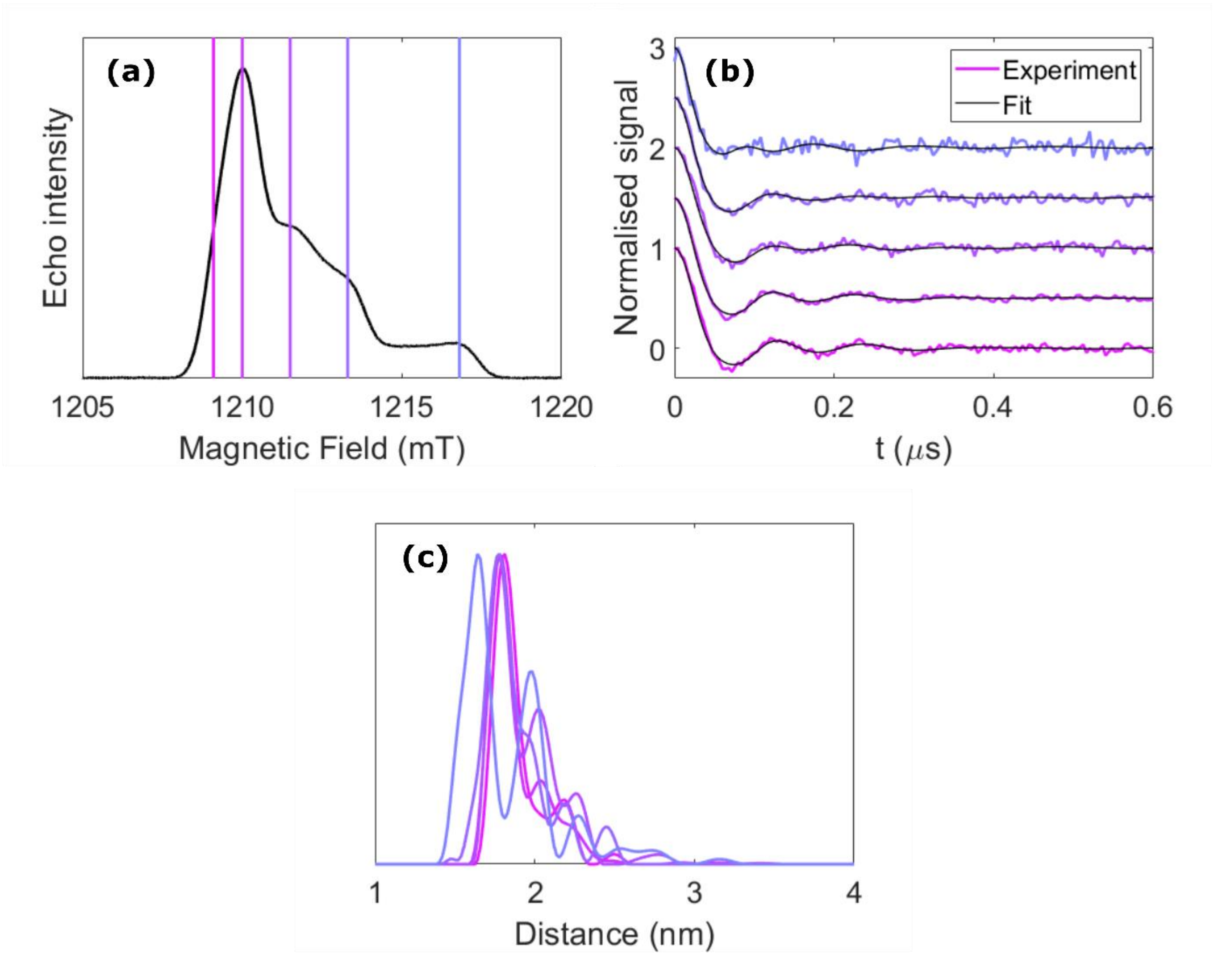

Figure S17. Orientation-independent analysis of individual dipolar traces. (a) Echo-detected field-swept spectrum in the dark, showing the field positions where ReLaserIMD traces were acquired. (b) Background-corrected and modulation depth-normalized ReLaserIMD traces (thick lines) and orientationally-independent fits by Tikhonov regularization using *DeerAnalysis2019* [2] (black), using a regularization parameter of 12.6. (c) Corresponding distance distributions.

S5. High-temperature experiments

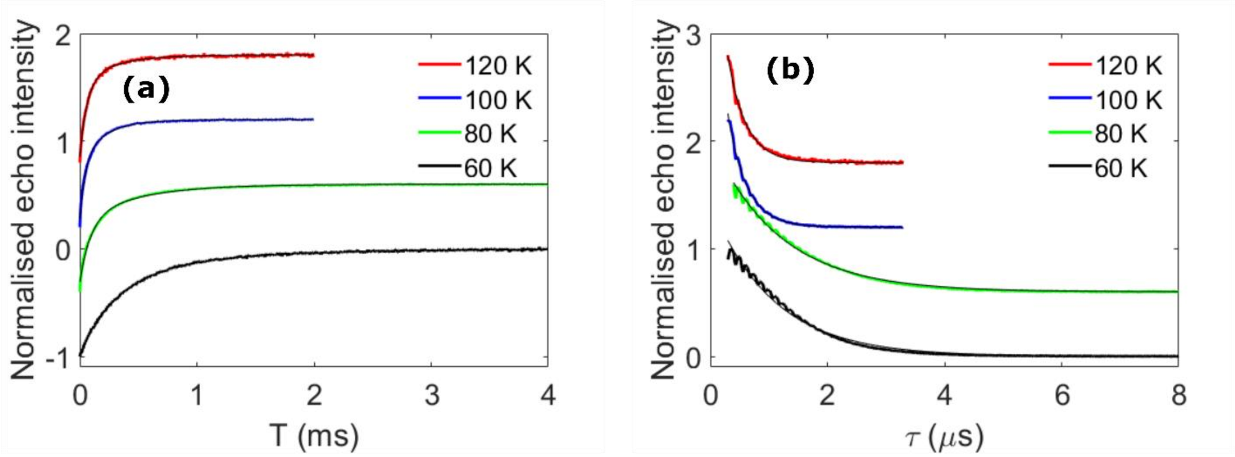

Figure S18. Characterization of the relaxation of the nitroxide spectral maximum in the dark at different temperatures: 60 K (black), 80 K (green), 100 K (blue) and 120 K (red). (a) Inversion-recovery experiments (thick lines) with biexponential fits (thin lines). The signal has been plotted as a positive decay for ease of analysis. (b) Phase-memory-time (T_m) experiments (thick lines) with monoexponential fits (thin lines). The lifetimes determined from the fits are reported in Table S1.

Table S1. Lifetimes extracted from the fits in Figure S18.

Temperature [K]	Inversion-recovery lifetimes [ms] (weights)	T_m [μs]
60	0.315 ± 0.008 (0.73)<br>1.03 ± 0.04 (0.27)	1.07 ± 0.01
80	0.094 ± 0.003 (0.64)<br>0.51 ± 0.01 (0.36)	1.15 ± 0.01
100	0.046 ± 0.002 (0.58)<br>0.243 ± 0.007 (0.42)	0.323 ± 0.003
120	0.061 ± 0.003 (0.73)<br>0.29 ± 0.02 (0.27)	0.309 ± 0.006

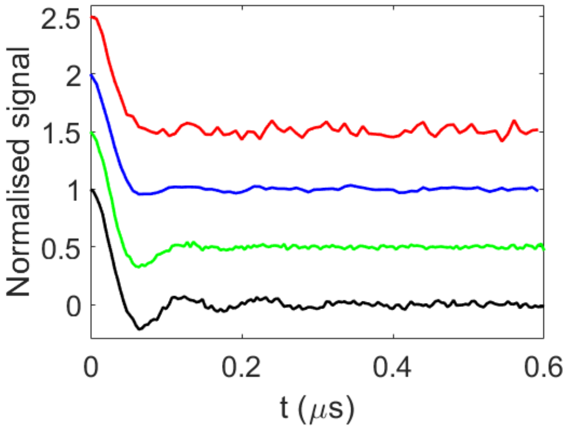

Figure S19. Background-corrected and modulation-depth normalized ReLaserIMD traces acquired at the nitroxide signal maximum, at different temperatures: 60 K (black), 80 K (green), 100 K (blue) and 120 K (red). The traces have been averaged for the following number of scans: 1875, 2520, 6960 and 29350, respectively. A time step of 8 ns was used to acquire the traces at 100 K and 120 K in order to reduce the acquisition time, while a 4 ns step size was used at 60 and 80 K. Modulation depths were ~ 7% before normalization in all cases.

REFERENCES

1. Stoll, S.; Schweiger, A. EasySpin, a Comprehensive Software Package for Spectral Simulation and Analysis in EPR. *J. Magn. Reson.* **2006**, *178*, 42–55, doi:10.1016/j.jmr.2005.08.013.

2. Jeschke, G.; Chechik, V.; Ionita, P.; Godt, A.; Zimmermann, H.; Banham, J.; Timmel, C.R.; Hilger, D.; Jung, H. DeerAnalysis2006 - A Comprehensive Software Package for Analyzing Pulsed ELDOR Data. *Appl. Magn. Reson.* **2006**, *30*, 473–498, doi:10.1007/BF03166213.
